# Nucleic acid-based therapy for coronavirus disease 2019

**DOI:** 10.1016/j.heliyon.2020.e05007

**Published:** 2020-09-19

**Authors:** Ravikant Piyush, Keshav Rajarshi, Aroni Chatterjee, Rajni Khan, Shashikant Ray

**Affiliations:** a School of Biotechnology, Madurai Kamaraj University, Madurai, Tamil Nadu 625021, India; b School of Community Science and Technology (SOCSAT) Indian Institute of Engineering Science and Technology (IIEST), Shibpur, Howrah, West Bengal 711103, India; c Indian Council of Medical Research (ICMR)—Virus Research Laboratory, NICED, Kolkata, India; d Motihari College of Engineering, Bariyarpur, Motihari, NH 28A, Furshatpur, Motihari, Bihar 845401, India; e Department of Biotechnology, Mahatma Gandhi Central University Motihari, 845401, India

**Keywords:** Microbiology, Biotechnology, Molecular biology, Epidemiology, Virology, Regenerative medicine, Covid-19, SARS-CoV-2, Nucleic acid based therapy, siRNA, Ribozymes, DNAzymes, Aptamers, Antisense, Oligonucleotide

## Abstract

The coronavirus disease 2019 (COVID-19), the pandemic that originated in China has already spread into more than 190 countries, resulting in huge loss of human life and many more are at the stake of losing it; if not intervened with the best therapeutics to contain the disease. For that aspect, various scientific groups are continuously involved in the development of an effective line of treatment to control the novel coronavirus from spreading rapidly. Worldwide scientists are evaluating various biomolecules and synthetic inhibitors against COVID-19; where the nucleic acid-based molecules may be considered as potential drug candidates. These molecules have been proved potentially effective against SARS-CoV, which shares high sequence similarity with SARS-CoV-2. Recent advancements in nucleic acid-based therapeutics are helpful in targeted drug delivery, safely and effectively. The use of nucleic acid-based molecules also known to regulate the level of gene expression inside the target cells. This review mainly focuses on various nucleic acid-based biologically active molecules and their therapeutic potentials in developing vaccines for SARS-CoV-2.

## 1 Introduction

The world has already witnessed many viruses causing disease outbreaks in various regions across the globe in the past two decades; such as Severe acute respiratory syndrome coronavirus (SARS-CoV) epidemic in 2002–2003 originated in China [1], Influenza A pandemic in 2009, first reported in Spain [2], Middle East respiratory syndrome (MERS) pandemic in 2012, first identified in Saudi Arabia and the current [3], Severe acute respiratory syndrome coronavirus 2 (SARS-CoV-2 or COVID-19) pandemic in 2019–2020, originating from China. SARS-CoV-2 consists of a positive sense (+) single-strand RNA genome [4, 5]. It belongs to the β-coronavirus sub-family along with SARS-CoV and MERS-CoV [4, 6]. The whole-genome sequence of SARS-CoV-2 consists of 29,903 nucleotides assigned with GenBank accession number MN908947 and the order of gene present was: replicase ORF1ab, spike (S), envelope (E), membrane (M) and nucleocapsid (N) in 5′ to 3′ direction of the viral genome [7, 8, 9]. WHO officially designated this new disease as coronavirus disease-2019, i.e., COVID-19. To date, the virus has infected more than 17 million people, and more than 680,000 people lost their lives across the globe [10].

This unprecedented global crisis has posed a significant challenge for the human and has brought forward our therapeutic limitations to fight against an unpredictable deadly virus. In recent years significant progress has been made in the understanding of viral gene functioning, viral genomics, and target-based drug designing which have stimulated the development of many therapeutic strategies capable of efficiently blocking viral gene expression [11]. Among them, the nucleic acid-based therapeutic molecules have shown significant potential as therapeutic agents with potential anti-viral properties [12]. These proposed anti-viral drugs, feature a versatile mode of action and has been designed to specifically arrest viral disease progression [13]. The nucleic acid-based drugs have been shown to elicit a broad spectrum of anti-viral immunity in the body along with suppressing viral replication and gene expression [14]. This phenomenon of producing an effective immunity is particularly important in their use against the development of nucleic acid based therapeutic drugs for the treatment of SARS-CoV-2.

By the introduction of nucleic acid-based therapeutic technologies, the gene expression can be regulated either at the transcriptional or post-transcriptional level [15]. Nucleic acid-based therapeutic molecules try to restore the homeostatic balance in two ways: overexpression of protective genes and silencing of damaged genes. Nucleic acid-based therapeutic biomolecules have also shown some promising results in pulmonary diseases [16]. Nucleic acid-based therapies, especially, RNA therapies including RNAi (RNA interference), siRNAs (small interfering RNA) and RNA aptamers, Ribozymes and ASOs (antisense oligonucleotides) target and neutralize the crucial components of the virus-like specific mRNA molecules, viral proteins like E (envelope), M (membrane), or N (nucleocapsid), or SARS helicase, etc. These biomolecules have also been found to be effective during the previous epidemic due to SARS-CoV [17]. The phylogenetic analysis of SARS-CoV-2 with SARS-CoV has shown that they share 89.1% similarities among each other [7, 8, 9], the use of nucleic acid-based molecules against SARS-CoV-2 may emerge as a potential therapeutic strategy.

Several viruses have been reported to show tropism towards the cells of respiratory tracts which not only help in the entry of virus particles inside respiratory tracts but it also helps to causes the infection in the host cells [13]. The physiological function of the lungs makes it highly exposed to pollutants and viral particles from outside [13]. Due to this reason, lungs get susceptible to several diseases ranging from common viral infection to lung cancer. Viruses that infect the respiratory tract spread rapidly among the population due to their simple and easy mode of transmission [13]. The transmission of such viruses mainly takes place either by physical contact with the infected person or by droplets containing the viral particles [13].

Currently, there is no therapeutic agent or vaccination for the treatment of SARS-CoV-2. In the present scenario, patients are administrated with a combination of drugs such as remdesivir, lopinavir, etc. [18], hydroxychloroquine, azithromycin, zinc sulfate and corticosteroids. Patients with severe infections are treated by collateral care like ventilation and fluid management. So, discovering a novel therapeutic approach that could help in the control, prevention, and treatment of SARS-CoV-2 infection is an urgent need to save the entire globe from this pandemic situations.

The objective of this review is to put forward the current scenario of the development and efficacy of nucleic acid-based therapeutics as potential anti-viral agents. Here, we have also summarized the potential benefits and challenges in the application of these anti-viral agents in the context of SARS-CoV-2 infection.

### 1.1 SARS-CoV-2 pathogenesis

The entry of virus particles inside the host cells determines the viral infectivity and pathogenicity [19]. The spike protein present on the surface of SARS-CoV-2 facilitates the entry of virus particles through the human angiotensin-converting enzyme 2 (hACE2) receptors binding domain [20] and is proteolytic activated by host cell proteases [19]. Therefore, several research groups are trying to decipher the interaction of hACE2 and spike protein as a novel drug target site to stop the pathogenicity [5, 21]. The hACE2 receptors present on the lungs, arteries, heart, kidneys and small intestine, colon, thymus, bone marrow, lymph nodes, the brain of the host cells [22]. The S1 subunit of the spike protein binds to the receptor-binding domain and assists the attachment of spike protein to the receptor, resulting in conformational changes into the spike protein [21]. TMPRSS211, a serine protease and lysosomal proteases cathepsins produced by the host cell cleaves the spike protein at the boundary of S1/S2 in such a way that S1 dissociates from the complex [19] and intense structural change in S2 domain was observed, which is necessary for the fusion of the virus into the host cell membrane [23, 24]. Membrane fusion and internalization of the virus are carried out via the S2 domain of the spike protein [5]. Unlike SARS-CoV, the pre-activation of SARS-CoV-2 entry inside the host cell is caused by proprotein convertase furin, which reduces its dependency on target cell proteases for invasion [19].

### 1.2 Immune response towards SARS-CoV-2 pathogenesis

Progression of the virus and failure of the immune system causes severe damage to the other parts of the body, especially those organs which express the hACE2 receptors highly, such as kidneys, lungs, and intestines [25]. The immune response towards any disease differs due to genetic variations from individual to individual. The SARS-CoV-2 infection can be classified into two categories: initial (non-severe) and latter (severe) [26]. In the initial stages, macrophages and granulocytes mediate the inflammatory responses. SARS-CoV-2 infection in the respiratory system has been reported to activate nuclear factor kappa-light-chain-enhancer of activated B cells (NF-κB) through pattern recognition receptors (PRRs), leading to the activation of pro-inflammatory cytokines, including interleukin-6 (IL-6), chemokines and tumor necrosis factors (TNFs) [27]. Signal transducer and activator of transcription 3 (STAT3) is required for the hyper-activation of NF-κB via activation of the IL-6 amplifier (IL-6 Amp), leading to autoimmune and multiple inflammatory diseases in the patients [28]. But in severe cases, a powerful chronic inflammation due to cytokine response from both immune and non-immune cells causes severe damage to the host or even death due to immune-mediated Adverse Drug Reactions [29, 30] (ADRs) [27].

## 2 Nucleic acid-based vaccines

Although lots of efforts are being put into research and development of DNA or RNA vaccine over the past few years, currently it has not been perfected enough to be used in humans [31]. Though several DNA vaccines have been accepted to be used for trials on animals [31]. Improvements in the DNA and RNA vaccine development strategy might turn out to be very crucial, keeping in view the increased frequency of epidemics, also efficient development of the vaccine may prevent infections caused by highly transmittable pathogens. Since synthetic DNA and RNA are easier to construct, therefore, DNA and RNA based approach could provide for more quick development of vaccines [32].

The nucleic acid-based vaccination technologies involve the use of RNA (mRNA) [33] or plasmid DNA, which encodes for antigen. These antigens encoded by the nucleic acid can trigger humoral as well as cell-mediated immune responses upon their expression after cellular uptake [34]. The nucleic acid-based vaccination technology is considered versatile and flexible as it allows easy maneuvering and manipulation of the antigen. The advantage of producing antigens in the target cells is that it imitates the protein synthesis during the infection as the protein remains localized in the plasma membrane, and protein modification processes like glycosylation can occur with great extents of fidelity [35]. Notably, they assist the delivery of an antigen of choice, irrespective of whether it was originated from a bacteria, parasite, or virus, thus facilitating the development of vaccines against a wide range of pathogens [31]. The different nucleic acid-based vaccine candidates which are in the different phase in clinical trails are described in Table 1.

**Table 1:** Nucleic acid derived vaccine candidates for COVID-19 in Clinical phase.

Vaccine candidate	Current status	Vaccine characteristics	Developer
mRNA-1273	Phase 1/Phase 2 (NCT04283461)	Lipid nanoparticle (LNP)-encapsulated mRNA-based vaccine. Pre-fusion stabilized spike (S) protein of SARS-CoV-2	Moderna
INO-4800	Phase 1 (NCT04336410)	DNA plasmid Vaccine encoding spike (S) protein delivered by Electroporation Device	Inovio Pharmaceuticals
ChAdOx1	Phase 1/Phase 2 (NCT04324606)	Non-replicating viral vector	University of Oxford
Pathogen-specific-aAPC	Phase 1 (NCT04299724)	Artificial antigen presenting cells (aAPCs) modified with lentiviral vector expressing synthetic minigene based on domains of selected viral proteins	Shenzhen Geno-Immune Medical Institute
LV-SMENP-DC	Phase 1 (NCT04276896)	Dendritic cells (DCs) modified with lentiviral vector expressing synthetic minigene based on domains of selected viral proteins; administered with antigen-specific cytotoxic T lymphocytes (CTLs)	Shenzhen Geno-Immune Medical Institute
Ad5-nCoV	Phase 1 (NCT04313127)	Adenovirus type 5 vector that encodes S protein	CanSino Biologicals

Reference: ClinicalTrials.gov.

Several nucleic acid-based molecules such as aptamers, siRNA, and miRNA have been used for the treatment of severe viral infections including HIV-1 (human immunodeficiency virus) [36, 37]. The infections caused by the H1N1 influenza A virus have been treated via the miRNA-based therapeutic molecules and have proved to be an effective medication [38, 39, 40]. Further, it has been reported that the ribozyme inhibited the infection of influenza virus both *in vitro* and *in vivo* [41]. In addition it was also found that modified ASOs (antisense oligonucleotides) were effective in suppression of the infection by influenza A/PR8/34 (H1N1) virus [42]. ASOs were also reported to be effective in the inhibition of RSV (respiratory syncytial virus) infection [43].

### 2.1 DNA vaccines

Incorporation of a eukaryotic expression cassette that encodes for single or multiple antigens of interest into a bacterial plasmid leads to the generation of DNA vaccines. The plasmid backbone is comprised of the origin of replication and the sequences for antibiotic resistance genes which is used as a selection marker. Mostly, these selectable markers include antibiotic resistance genes against antibiotics like kanamycin [29]. DoggyboneTM (covalently closed linear DNA construct) [30] and Minicircle DNA [31], comprised of the gene expressing cassette devoid of the backbone of bacterial plasmid DNA. Several studies have reported that bacterial backbone resulted in a lower level of reporter transgene expression compared with mice receiving the expression cassette alone [44, 45]. The difference in the expression level may be due to the formation of large random concatamers and smaller circles by the linear DNA than the closed-circular DNA (ccDNA) which remained as circular structures [44]. It was hypothesized that the low expression of the transgene is due to the inhibition resulted by the covalent attachment of the bacterial backbone to the expression cassette [45].

DNA vaccines were most commonly administered through intradermal (ID) or intramuscular (IM) route with the aid of a conventional needle, which resulted in very low immunogenicity [44]. Hence, in order to enhance the DNA uptake, immunogenicity, and expression, several methods have been developed which involve the use of devices like gene gun, *in vivo* electroporation, and jet injections (needle-free). These devices have displayed assuring results in both clinical and pre-clinical trials [46, 47].

#### 2.1.1 DNA vaccines: mode of action

Several studies suggest that DNA vaccines induce both cellular and humoral immune responses via the activation of CD4^+^ helper T-cells and CD8^+^ cytotoxic cells [48, 49]. DNA vaccines are recognized by various immune receptors upon their entry inside the cell [50]. Previous experimental studies involving the ID administration of DNA coated gold particles suggested transfection in keratinocytes as well as in the professional antigen-presenting cells, i.e., Langerhans cells. The MHC class I and class II-restricted recognition of antigen by CD8^+^ cytotoxic and CD4^+^ helper T-cells was explained via this transfection [51]. The intramuscular administration of DNA vectors, however, resulted in the transfection in myocytes [52]. The function of APCs derived from bone marrow, in the activation of MHC class I restricted CD8^+^ cells upon DNA vaccination, has been well documented by several studies [53, 54, 55]. Cross-priming and presentation of both MHC class I and class II-restricted antigens by profession APCs upon phagocytization of transfected somatic cells is the most probable mechanism of action in the case of DNA vaccination [56].

### 2.2 RNA vaccines

mRNA carries the genetic information as an intermediate, which can be used as a template for protein production in the vaccinated subject, endogenously [57]. Non- replicating mRNA and Self-amplifying mRNA are the two major types of RNA that have been used so far as prophylactic vaccines against infectious disease-causing pathogens [58]. The sequence of the specific antigen is flanked by the 5′ and 3′ untranslated regions (UTRs) in the non-replicating mRNA [58]. The advantage of non-replicating mRNA over self-amplifying mRNA is that they have a small size, simpler construct, and lack any extra encoded proteins, which could trigger an unwanted immune response [59].

The mRNA based vaccine needs an efficient delivery and expression into the cytoplasm but the plasma membrane hinders the entry of mRNA [60]. Further, intramuscular delivery leads to a relatively low cellular and humoral response [61, 62, 63, 64]. Hence, direct injection of naked mRNA can be executed through intranodal and intradermal administration in order to efficiently target the APCs [65, 66].

#### 2.2.1 RNA vaccines: mode of action

Several innate immune receptors (cytosolic and endosomal) and cell surface receptors recognize the exogenous mRNA, thus making it immunostimulatory [67]. Various pattern recognition receptors (PRRs) like TLR3, TLR7, and TLR8 situated in endosomes and MDA-5, PKR, RIG-I, NLRP3, and NOD-2 present in the cytoplasm of mammalian cells assist in sensing the foreign RNA [68, 69, 70]. mRNA vaccine-induced activation of PRRs leads to a vigorous immune response resulting in the production of cytokines and chemokines such as TNF and IL-12 at the site of inoculation [71]. mRNA immunization administered intradermally leads to the upregulation of the expression of various chemokines, including CXCL10, CXCL11, and CXCR3-ligands CXCL9 [69]. These chemokines assemble the innate immune cells like macrophages and dendritic cells at the site of injection [71].

### 2.3 RNA interference and nucleic acid-based therapeutic molecules

RNA interference (RNAi), a primitive mechanism of gene regulation, plays a crucial role in the control of gene expression in all eukaryotes [72]. Double-stranded RNA molecules are used to silence the post-transcriptional expression of homologous target genes during RNA interference, which is an evolutionarily conserved phenomenon [73]. In the late 1980s, this mechanism was first discovered in plants, and then in the year 1988, it was found to be occurring in *Caenorhabditis elegans* [74, 75]. A similar process was demonstrated in the mammalian cells (in 2005), which resulted in the development of new and enhanced tools to study and better understand the function of the gene [76, 77]. RNAi is a mechanism that is associated with the innate immune response in order to protect the cells from the attack of nucleic acids belonging to pathogens such as bacteria or viruses [78].

Several RNA interference-based therapeutic approaches have been found to treat many of the pulmonary diseases [79, 80]. Some of the molecules that have been extensively experimented in the past include antisense oligonucleotides, aptamers, siRNA, miRNA, etc [81]. These therapeutic molecules have shown some promising effects against the viral infection in the respiratory tract and to several other diseases [82]. In a study conducted on the cultured Vero cells (kidney epithelial cells) obtained from African green monkeys, inhibition of replication of virus along with cytopathic attenuation effect was demonstrated upon administration of synthetic siRNAs [17]. In particular, a strong inhibition in the replication of the virus was exhibited by the siRNAs, which targeted the *S* (Spike) sequence. The *S* gene was also proven to be a good target for inhibition of SARS-CoV in the cultured cells via expressed RNAi activators [83]. RNA interference has also been proven efficient in combating the influenza virus [84] and RSV via targeting their mRNA [85, 86]. Some RNAi patents related to previously encountered coronaviruses, i.e., SARS and MERS have been enlisted in Table 2 [87].

**Table 2:** RNAi patents related to previously encountered coronaviruses [87].

Virus	Type of RNAi	Target of action	Patent Number	Developing Organization
SARS-CoV	siRNA	RdRP (RNA-dependent RNA polymerase)	CN101113158	Sichuan University
MERS-CoV	siRNA	Spike protein, RdRp, PLpro	WO2017044507	Sirnaomics, Inc.
SARS-CoV	RNA aptamer	nucleocapsid	KR2012139512	Kookmin University, Industry-Academic Cooperation Foundation
SARS-CoV	siRNA	S,N,M,E gene, Replicase A1	US20050004063	The University of Hongkong
SARS-CoV	siRNA	*orf*3*a*	CN101085986	Shanghai Institutes for Biological Sciences, Chinese Academy of Sciences
SARS-CoV	siRNA	M gene	CN101173275	Institute of Basic Medical Sciences, Chinese Academy of Medical Sciences
SARS-CoV	Modified oligonucleotide (ASO)	Various Regions	WO2005023083	Isis Pharmaceuticals, Inc.

#### 2.3.1 Aptamers

Research on viruses like adenovirus or HIV resulted in the discovery of a new class of small RNAs, in the 1980s, called aptamers [88]. These are short oligonucleotides (RNA or DNA) that specifically recognize and bind to the target sites, with a unique 3-D structure. It can be used as a targeting moiety for drug delivery or as inhibitors of protein function [89, 90]. These molecules were first described as the TAR-aptamer (trans-activation response), a virus-encoded transcript in HIV-1 [91, 92]. The TAR aptamer was found to bind with the Trans-Activator of Transcription (Tat) protein of the virus [92]. It was revealed that the complex formation of this aptamer inhibited the function of Tat protein, which was essential for viral replication as well as regulation of gene expression of both cellular and viral gene [93]. The binding of aptamers to their respective target is highly specified, and the order of affinity for the target site is similar to that of the monoclonal antibodies [94]. Most of the therapies involving the use of oligonucleotides destroy the mRNA by targeting the translational machinery of the cell, whereas, aptamers directly target the proteins and transform or alter their function by binding to them [95].

#### 2.3.2 Small interfering RNAs (siRNAs) and micro RNAs (miRNAs)

Small interfering RNAs (siRNAs) are 21–23 base pairs long double-stranded molecules, which is used for silencing the target genes in a sequence-specific manner [96]. Whereas, micro RNAs (miRNAs) are 18–24 nucleotides long single-stranded endogenous non-encoding RNA molecules that are used as key regulators for various cellular functions [97]. Both siRNAs and miRNAs can interact with the multifunctional protein, Argonaute-2, and associate into the RNA-induced silencing complex (RISC) [98]. The site-specific gene silencing effect of siRNAs enables it to be used as an indispensable tool for targeting the expression of the gene of interest. The therapeutic approaches involving siRNAs are more specific than miRNAs- mediated therapeutics as miRNAs have the ability to hybridize with mRNA having partially complementary sequences [99, 100].

#### 2.3.3 Ribozymes and DNAzymes

Several essential biological processes, such as the replication of RNA genome, RNA processing, RNA silencing, and peptide bond formation during translation has been described by the chemical catalysis of RNA [101]. Thus, RNA molecules with catalytic activities can be referred to as ribozymes [101, 102, 103, 104]. RNA-RNA interaction between the ribozymes and its substrate molecules determines the highly sequence-specific reactions catalyzed by ribozymes [105]. Thus, the capacity of ribozymes to inactivate other RNA molecules in a specific manner has encouraged its use as a potential gene suppressor and promising molecular tools with various applications [105]. In order to cleave substrate mRNA in a sequence-specific manner, nucleic acid enzymes like ribozymes or deoxyribozymes are used [106]. This results in a specific blockage of expression of detrimental genes. In lower eukaryotes, some bacteria and viruses, several ribozymes promoting the intermolecular splicing, or catalyzing the cleavage reaction have been found (Reference) [107].

Deoxyribozyme (Dz) or DNA enzyme are synthetic single-stranded DNA particles that have the potential ability to recognize and cut the particular mRNA molecule at a specific position [108]. Dz contains one catalytic domain flanked by two variable arms which are complementary to the targeted mRNA. When both arms of Dz bind with the complementary targeted mRNA sequences according to Watson and Crick pairing, catalytic domain help to cleave the targeted mRNA sequences [108]. They are essentially the analogs of ribozymes [109]. In ribozymes, the ribonucleotide motifs essential for catalytic activity are often biologically unstable. To overcome this, in DNAzymes, these motifs are replaced with stable DNA molecule. This provides enhanced biological stability and more synthetic options in producing modified DNAzymes [110, 111, 112, 113, 114].

#### 2.3.4 Antisense oligonucleotides (ASOs)

ASOs are single strands of DNA or RNA that modifies the function of mRNA through complementary binding [81, 115]. The modified ASO was first used by Matsukura and coworkers, for the blockage of HIV replication [116].

The hybridization triggers diverse mechanisms that could result in up-regulation or down-regulation of gene expression [81]. It can also interfere with RNA function, such as blocking mRNA association with specific transcription factors and by inhibiting RNA-mediated telomerase activity, etc. [117, 118, 119]. Zamecnik and Stephenson discovered the prospective of oligonucleotides to function as antisense agents that hinder the replication of viruses in cell culture [120]. Around 15–20 nucleotides are present in these oligonucleotides, which are complementary to their target mRNA. This strategy can play a crucial role in the field of drug discovery. Usually, ASOs are developed in order to activate RNAase H that further cleaves the RNA moiety of an RNA-DNA heteroduplex and results in degradation of the target. Another mechanism by which this antisense technology works is by restricting the binding of either the ribosome or the polymerase to the 5′ terminus of the target sequence, thus inhibiting the translation by preventing the congregation of the target machinery.

#### 2.3.5 Peptide nucleic acids (PNAs)

In 1991, PNAs was first time introduced by Nelson et, al as a molecular biology tool [121]. It is an artificial imitator of DNA in which the phosphate backbone is swapped with pseudo peptide polymers linked with nucleic acid bases as a side chain [122]. The polypeptide backbone is mainly made up of N-(2-aminoethyl) glycine which is linked with nitrogenous bases of nucleic acids through methyl carbonyl linker [122]. PNAs cannot be degraded either by DNAse or protease which extends the lifetime of PNAs inside the cells [123]. Lack of phosphate backbones makes PNAs chargeless which enhances the specificity, stability and higher affinity of PNAs towards DNA/RNA without causing any repulsion, unlike other nucleic acid hybridization methods [123, 124]. Since, PNAs have a high affinity towards binding DNA or mRNA thereby it regulates the targeted gene expression at the level of transcription and translation [123]. These days this technique is used in molecular biology as a diagnostic assay and antisense therapy.

## 3 Evidence supporting the nucleic acid-based therapeutic strategy

In research carried out by Wang et al. in 2004, has shown that RNA interference (RNAi) activators are effective against SARS-CoV on Vero cells by inhibiting the replication of SARS-CoV [125]. In the study, the effect of six different vector-based siRNAs was analyzed for the inhibition of replication of SARS-CoV [125]. It was found that only two of them efficiently blocked the viral replication in Vero cells by targeting the RNA polymerase, thus suggesting that anti-SARS agents could have developed by using the siRNA [125]. In another study, several potential inhibitors that target particular steps of the life cycle of the coronavirus were tested in order to investigate the therapeutic options [126]. The specific steps of the life cycle of the coronavirus that was target by these potential inhibitors were membrane fusion, binding to the receptor, translation, post-translation processing, transcription, and release of the virus [127]. HCoV-NL63 S glycoprotein, a viral entry protein, was found to be the target site of designed siRNA. In virus-infected cells, these designed siRNAs significantly inhibited the infection [128]. In another study, the synthesis of chimeric RNA-DNA hammerhead ribozyme targeting SARS-CoV was done. In order to ensure its activity, in vitro cleavage reactions were performed using the synthesized ribozyme. The results suggested that ribozymes (Rz) were found to be useful in inhibiting viral replication up to 60% by using chimeric DNA-RNA hammerhead ribozyme. 3T3 cells (fibroblast cell line) transfected with a recombinant plasmid when targeted using chimeric construct significantly reduced the expression of SARS-CoV RNA. Thus found to be a potent and feasible treatment option for SARS [129].

In the mice models and Vero E6 cell line, Dz has been reported to inhibit the replication of several viruses like the influenza virus, respiratory syncytial virus (RSV), and SARS-CoV by specifically targeting the vitals genes which are responsible for replication of these viruses [108]. It has been reported that a mono Dz-104 possessing 10–23 catalytic motifs targeted 5′ untranslated regions (UTR) which are highly conserved in the SARS-CoV genome and thereby suppressed SARS-CoV replication in Vero E6 cell line [130]. In the above study, the Vero E6 cell was co-transfected with p5′UTR-eGFP along with the various concentration of Dz and incubated for 12 h (hr) in the presence of lipofectamine plus. The effect of the varying concentration of Dz was observed by fluorescence microscopy by using the eGFP fluorescence [130]. Ahn et al. evaluated cells transfected with SARS-CoV replicon, showed significant suppression of SARS-CoV replication upon treating with peptide nucleic acid (PNA) (50% inhibitory concentration of 4.4 μM) fused with cell penetrating-peptide. The antiviral effects of antisense peptide nucleic acids (PNAs) were used to targeting a highly conserved RNA sequence on the programmed −1 ribosomal frameshifting (– PRF) signal in the SARS-CoV virus. The PNA that was bound to the specific sequence at the pseudoknot structure in the −1 PRF signal and thereby inhibited the ribosomal frameshift [131]. Nucleocapsid (N) protein of SARS-CoV is one of the most abundant target proteins for developing aptamers. Jang et al. used RNA aptamer ES15 to target nsP10 (nonstructural Protein 10) showed inhibiting viral replication in dose-dependent manner (IC_50_ = 1.2 nM) [132].

## 4 Nucleic acid-based vaccine development platforms and patents

Various major vaccine development platforms are advancing with their nucleic acid-based therapeutic models towards its clinical testing and evaluation [58]. The prime characteristics of the vaccine development include the cost, flexibility, speed, immunogenicity, reactogenicity, and safety of the manufacture [133]. The vaccine stability and durability are also of prime concern in vaccine development. Currently, several companies like Moderna, Inovio, BioNtech, and Curevac have taken initiatives to develop a potential therapeutic strategy against COVID-19, which involves the use of nucleic acid molecules [134]. Moderna Inc. USA, Inovio Pharmaceuticals, USA and Pfizer in collaboration with BioNTECH, has selected mRNA-1273, INO-4800 DNA vaccine and BNT162 mRNA vaccine, respectively as vaccine candidates for trial against COVID-19 [134].

### 4.1 DNA vaccines for SARS-CoV-2

**Figure 1:**
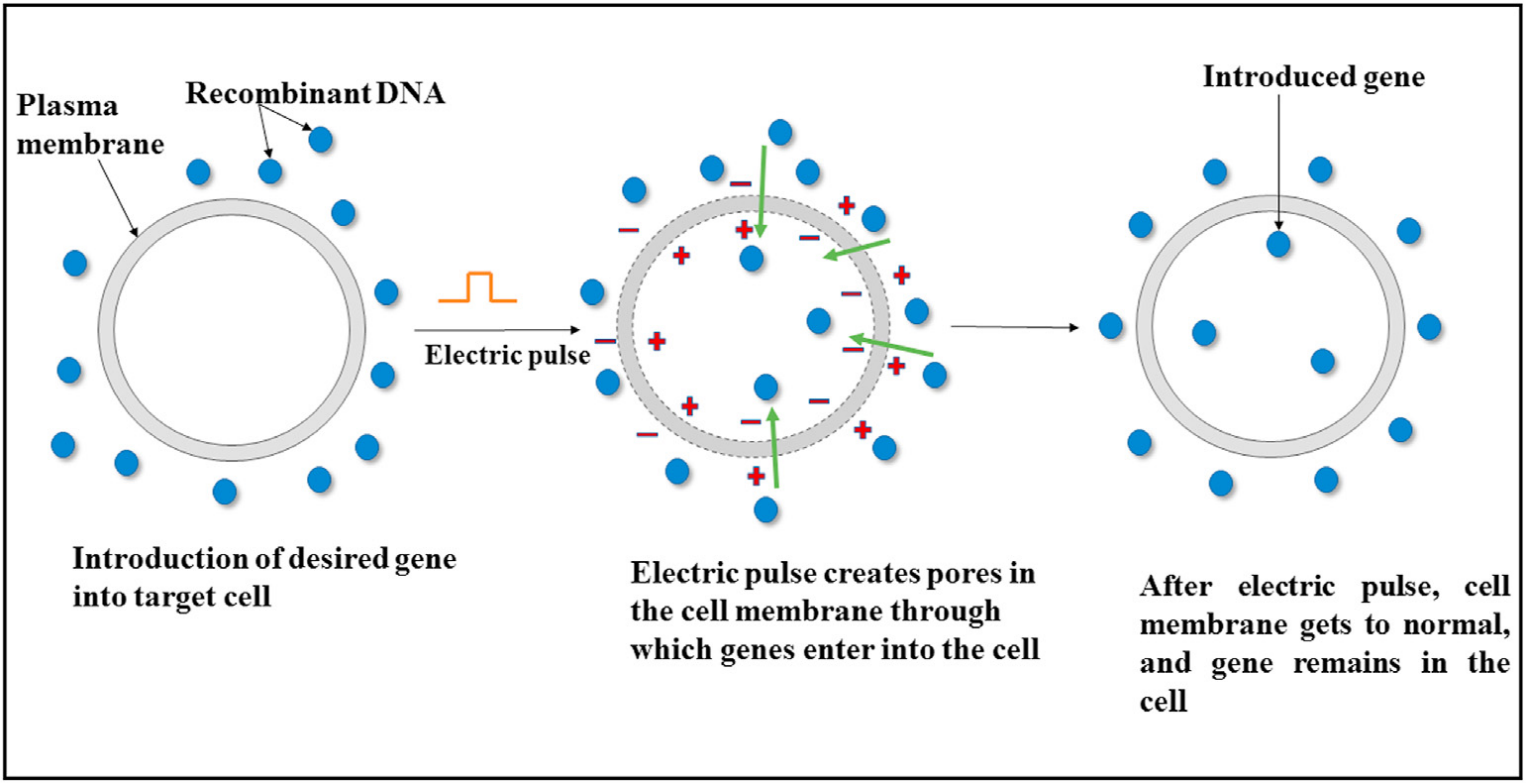
Schematic representation of electroporation mediated gene transfer. The desired gene is introduced into the target cell by applying an electric pulse to it. The electric pulse generates electric pores into the cell membrane through which the desired gene gets into it. As soon as the electric pulse is removed, the gene remains into the cell as the electric pores are closed and the cell membrane is in normal state. A similar method has been adopted for the delivery of INO-4800 into the host.

Developers like Takis, Karolinska Institute, and Inovio Pharmaceuticals are trying and testing various entry mechanisms for the DNA vaccines, including the needle injection and electroporation. Immunomic therapeutic and Osaka University are trying to develop a needle-free system for the delivery of DNA vaccines [136, 137]. INO-4800 developed by Inovio Pharmaceuticals is trying to use electroporation mediated gene transfer (Figure 1). The vaccine candidates of Takis and Karolinska Institute shall be administered with the intramuscularly. The Inovio Pharmaceuticals, Actranza™ lab and Pharmajet Tropis will be using a Needle-Free Injector System to deliver the vaccine through the intradermal route. The Immunomic Therapeutics, Inc selected SARS-CoV-2 DNA vaccines on the basis of their speculated ability to induce a potent immune response, and it will be ligated to the gene encoding the lysosomal-associated membrane protein (LAMP) for its successful delivery [137].

### 4.2 RNA vaccines for SARS-CoV-2

The mRNA-based vaccine has emerged out to a hopeful choice against SARS-CoV-2 for the development of the vaccine [138]. The mRNA based vaccines possess a beneficial edge over other biomolecules as it lacks the ability of integration into the genome, has the ability to induce autoantibodies, lack of persistence over time, their high purity and can be produced in large quantity to meet the need of vaccination [139]. Moderna Inc. The USA, and Pfizer in collaboration with BioNTECH has selected mRNA based vaccine candidates against SARS-CoV-2. Arcturus Therapeutics and Imperial College London are utilizing self-replicating or self-amplifying mRNA [140, 141], Translate Bio and Curevac are using optimized mRNA sequences which are unmodified [142, 143], while BioNTech, at present, is yet evaluating its three different RNA formats derived from different antigenic regions of spike glycoprotein [144]. Most of the companies are expected to target the major spike protein (structural protein) as the gene of choice, but all developers have not clearly stated so [145]. BioNTech offers three platforms for lipid-based delivery systems, i.e., lipoplexes, LNPs (lipid nanoparticles), and polyplexes [146]. The LUNAR system of Synthetic Genomics, which is the delivery platform to be used by Arcturus Therapeutics, seems to be widely applicable for several target tissues through multiple target routes [141].

#### 4.2.1 mRNA-1273 vaccine: a possible hope against COVID-19

One of the first vaccine, mRNA-1273, developed by the National Institute of Allergy and Infectious Diseases in association with Moderna, is under phase1/phase 2 of the clinical trial. An mRNA-1273 encoding for S protein of SARS-CoV-2 is encapsulated into lipid nanoparticles and delivered into the cell to generate an immune response against S protein [138].

**Figure 2:**
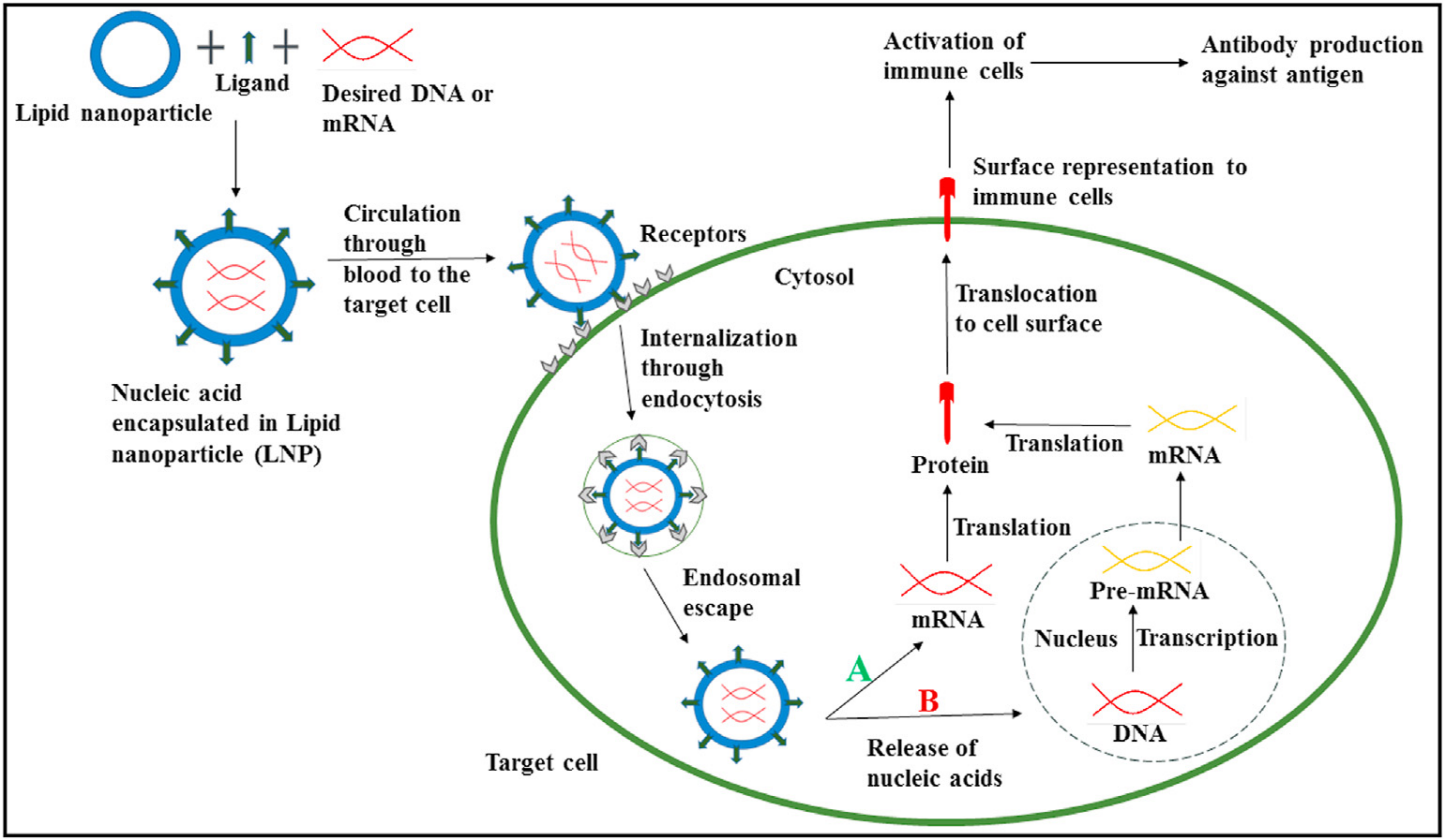
Schematic representation of lipid nanoparticle-mediated gene delivery. The nucleic acid can be either DNA or mRNA. A) In case of mRNA-1273, mRNA encoding pre-fusion spike protein is encapsulated in lipid nanoparticle. It is administrated intravenously into the patient. The ligand on lipid nanoparticle helps it to bind to the receptor and gets internalized through endocytosis. It is then, released into the cytosol where the gene enclosed in it gets released. mRNA is then translated into protein and the protein is translocated to the cell surface where it activates the immune cells to produce antibodies against it. B) If the nucleic acid is DNA, then it first goes into the nucleus to get transcribed into mRNA. In the cytoplasm, mRNA gets translated into the protein and the protein helps to generate an immune response in a similar way as stated above.

Using lipid nanoparticles (LNPs) as a carrier, the mRNA sequence of the recombinant target protein is delivered to the somatic cytoplasm for direct translation and encoding of the target protein [147] (Figure 2). The antigen-presenting cells quickly recognize these target proteins when released from the host cell [148]. Processing of these target proteins and their presentation is an important step for subsequent activation of both T and B cells resulting in cytotoxicity and humoral responses [148].

The therapeutic effect resulting from the antibodies against S protein may include the clearance of viral load from the infected cells, and reduced bioavailability of hACE2 might reduce the proliferation and spread of SARS-CoV-2 [149].

The inherent ratio of nucleotides to S protein (n/s) and outer surface protein to SARS-CoV-2 virion (s/v), both the ratio will play a crucial role to determine the amount of IgG antibody titer to be present and the number of booster doses to be given, to effectively neutralize the virus in the body [138]. A previous study on HIV has shown that low spike density with large spacing among them is incapable of activating B cells [150, 151].

## 5 Clinical trials

Globally, the ongoing clinical trials against COVID-19 is carried out at tremendous speed and scale. The fundamental and earlier pathways of vaccine development generally use to take a time scale of 10 years for development. This scenario has changed significantly during the pandemic of Ebola, reducing the time scale to nearly 5 years. At present, several vaccines are pipelined in clinical trials, and several vaccines are in pre-clinical trials.

**Figure 3:**
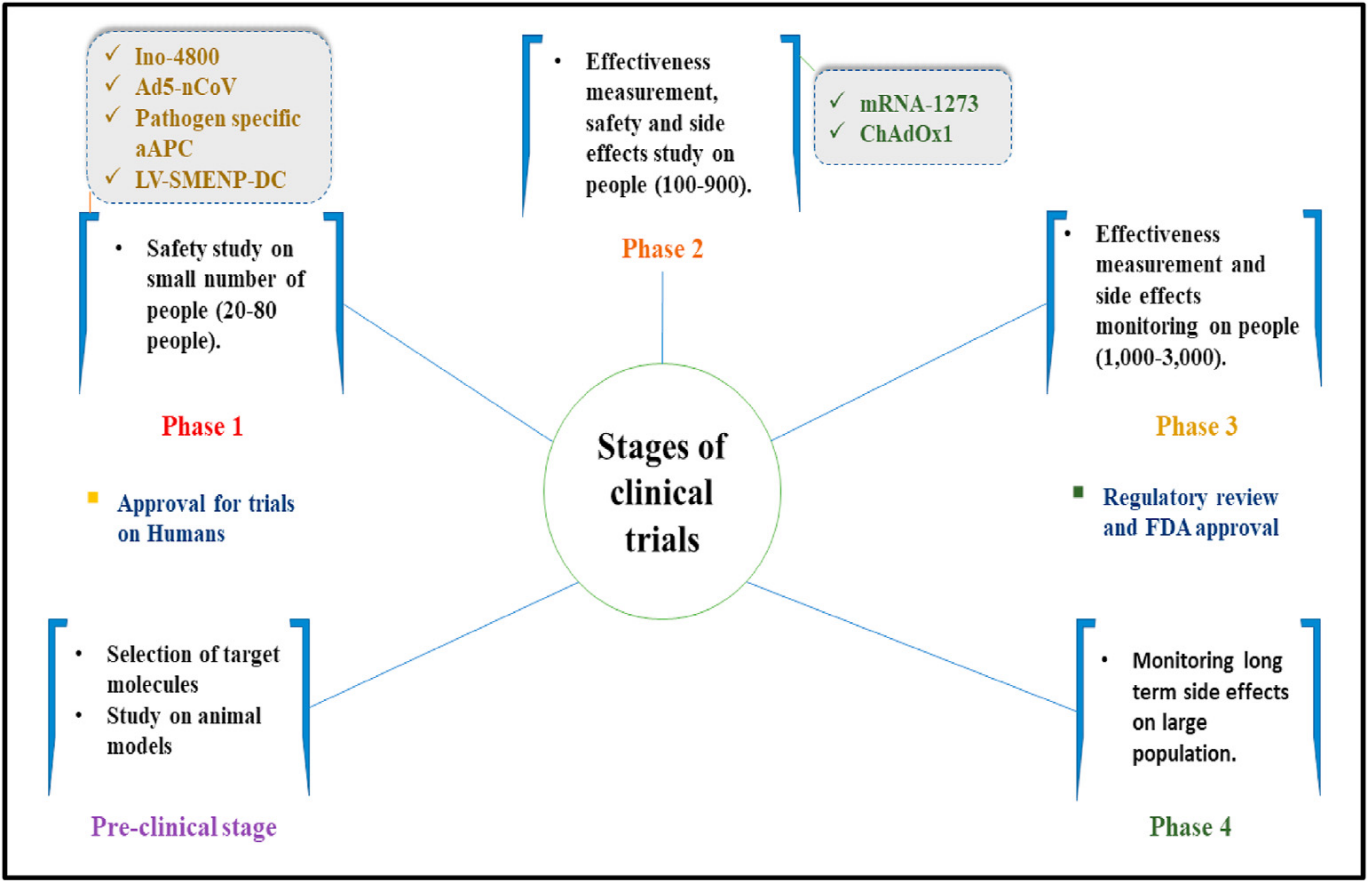
Schematic representation of different stages of clinical trials through which a drug or vaccine should pass to make it available for public use. The figure depicts the stages of clinical trials and vaccines that are in different phases of trials for the treatment of COVID-19.

According to WHO and clinicaltrials*.*gov, INO-4800, a DNA vaccine under the title “Safety, Tolerability and Immunogenicity of INO-4800 for COVID-19 in Healthy Volunteers” (trial identification number: NCT04336410) and mRNA-1273, an RNA vaccine under the title “Safety and Immunogenicity Study of 2019-nCoV Vaccine (mRNA-1273) for Prophylaxis of SARS-CoV-2 Infection (COVID-19)” (trial identification number: NCT04283461), are in phase-1 and phase 1/phase 2 of the clinical trials respectively and are showing promising effects against SARS-CoV-2 infection. These vaccines are targeting the spike (S) protein of novel coronavirus, which plays a promising role in the binding of the virus to the hACE2 receptors. Another RNA vaccine under the title “Study to Describe the Safety, Tolerability, Immunogenicity, and Potential Efficacy of RNA Vaccine Candidates Against COVID-19 in Healthy Adults” (trial identification number: NCT04368728) is in phase 1/phase 2 of the clinical trial (Figure 3).

## 6 Cost associated with vaccine development and production

Based on the drugs approved by FDA between 2009 to 2018, the mean research and development investment required to bring a new drug to the market was estimated to be $1336 million (M), and the median was estimated to be $985M [152]. The major components of cost driver include product development, facilities and equipment, direct labor, overhead, and licensing and commercialization [153]. In product development, the estimated cost range >500M USD with the risk-adjusted cost of 135–350M USD. In facilities and equipment, the estimated cost range between 50 to 700M USD. Direct labor and overhead cost range has been estimated to be 25% less than of total manufacturing costs, and up to 45% of labor and raw materials cost combined together respectively. For licensing and commercialization, the WHO process 300 (thousand) K USD for a site audit, 25K–100K USD for evaluation and annual fees of 4.8K–140K USD [154, 155, 156]. mRNA-1273 being developed by Moderna has received funding of $483M in April and $472M in July with total funding of $955M approximately whereas another leading vaccine, ChAdOx1 being developed by University of Oxford has received funding of £84M approximately [159].

In terms of the global distribution of vaccine production, there is a huge gap between developing nations and industrial nations. According to a survey conducted in 2015, only 5% of vaccines were distributed in about half of the world population in regions like Africa, Southeast Asia, and Eastern Mediterranean during the influenza pandemic in 2009 [160]. Based on this finding, the major challenge to the world after vaccine development will be of vaccine distribution across the globe.

### 6.1 Advantages and disadvantages of mRNA and DNA-based vaccines against SARS-CoV-2

The high potency of mRNA vaccines with only one or two low dose immunization is capable of generating potent antiviral neutralizing antibodies by activating both CD4^+^ and CD8^+^ T cells [161, 162, 163]. The structural modification of mRNA results in higher immunogenicity by improving its stability and translation efficacy [161, 162]. The potential risk of infection and insertion induced mutagenesis are minimized by mRNA based vaccines due to its natural degradation in cells [164]. In order to treat large populations, engineered mRNA facilitates the large-scale production of the required vaccine dose [165, 166].

The mRNA vaccination may be detrimental due to local and systemic inflammatory responses, possible development of autoreactive antibodies, persistence, and bio-distribution of induced immunogenic responses and toxic effect of delivery system components and non-native nucleotides [167, 168, 169]. Fatigue, chills, headache, myalgia, and pain at injection site are some of the solicited systemic and local adverse effect that occurred in more than half participants on which vaccination trail of mRNA-1273 was carried out [170]. Mutation in the spike protein increases the possibility that the vaccine will not be very effective in the long term [138].

Like mRNA vaccines, DNA vaccines generate effective antiviral neutralizing antibodies by activating CD4^+^ and CD8^+^ T cells [171, 172, 173]. The DNA drug product is stable for a longer duration and can be deployed in an effective and executable manner [174]. To meet the large demand to treat patients across the globe, the DNA plasmid manufacture process facilitates the scaled manufacture of the drug [173, 174].

The major cons associated with DNA vaccines were reported more prominent in humans and other large animals rather than small animal models [174]. It have been reported that in human and large animals DNA vaccine causes lower immunogenicity in comparison to inactivated vaccines, autoimmune responses, and DNA integration in the host genome, etc. [173, 175].

## 7 Conclusion

In current situations, every day a large number of the population is being infected from SARS-CoV-2. The need of the hour is the development of a novel therapeutic approach to confront the global crisis. Nucleic acid-based therapy is a novel therapeutic approach and has shown assuring results against SARS-CoV; therefore, its use is being explored for COVID-19. mRNA-1273 and INO-4800 are showing promising results in clinical trials in treating SARS-CoV-2 infection. Recent advancements in technology have provided better ways for specific delivery of these active biomolecules to the target cells without producing side effects. Several nucleic acid-based vaccine against SARS-CoV-2 are in the primary stages in the clinical trials.

In summary, it can be concluded that these nucleic acid-based therapeutic agents have proved to be a potent and versatile group of antiviral drugs that have already shown potential efficacy against several viruses including SARS-CoV and other chronic diseases. In-vitro and in-vivo studies have proclaimed their importance as promising antiviral agents. These nucleic-acid based inhibitors, when fully developed as drug molecules it may use against SARS-CoV-2 which can be an effective line of treatment for various other viral diseases.

## Declarations

### Author contribution statement

All authors listed have significantly contributed to the development and the writing of this article.

### Funding statement

This research did not receive any specific grant from funding agencies in the public, commercial, or not-for-profit sectors.

### Competing interest statement

The authors declare no conflict of interest.

### Additional information

No additional information is available for this paper.
